# A Case of Sudden Unexplained Death in Alcohol Misuse in a 49-Year-Old Woman

**DOI:** 10.7759/cureus.95849

**Published:** 2025-10-31

**Authors:** Jonathan Taube

**Affiliations:** 1 Medicine, Morriston Hospital, Swansea, GBR

**Keywords:** alcohol-related mortality, case report, liver cirrhosis, multi-organ failure, sudden unexplained death of alcohol misuse

## Abstract

A 49-year-old woman with a complex history of alcohol-related liver disease and multiple previous episodes of pancreatitis presented with acute gastrointestinal symptoms and rapidly deteriorated in the emergency department. Despite appropriate initial management, she became progressively hypotensive and hypoglycaemic and died within hours of admission. Post-mortem examination revealed multi-organ pathology, including hepatic steatosis, gastric mucosal inflammation, and acute tubular necrosis of the kidneys. No discrete anatomic cause of death was identified, and the post-mortem diagnosis was multi-organ failure secondary to sudden unexplained death in alcohol misuse (SUDAM) as the cause of death. This case highlights the complex interplay between chronic alcohol use, poor nutritional status, and sudden unexplained death, emphasising the importance of comprehensive post-mortem investigation in such presentations.

## Introduction

Alcohol misuse is a major global health burden, contributing to over three million deaths annually, representing nearly 6% of all global deaths [1]. Chronic alcohol consumption is associated with multisystem pathology, including alcoholic liver disease, pancreatitis, cardiomyopathy, and neurological disorders. Beyond these well-recognised complications, a subset of deaths occur suddenly and unexpectedly in individuals with alcohol misuse without an obvious anatomical or toxicological cause and are increasingly recognised under the entity of sudden unexplained death in alcohol misuse (SUDAM) [2].

SUDAM shares some clinical and pathological features with sudden arrhythmic death syndrome (SADS), particularly the unexpected nature of death in individuals without identifiable structural heart disease. However, while SADS is typically attributed to primary cardiac arrhythmias arising from inherited or acquired electrical abnormalities, SUDAM is thought to result from a multifactorial interplay of chronic alcohol-induced organ damage, metabolic disturbances, and nutritional deficiencies [2,3]. SUDAM differs from SADS in its underlying mechanisms, reflecting systemic alcohol-related pathology rather than isolated cardiac electrophysiological dysfunction. Recognition of SUDAM is important, as it may account for a significant proportion of unexplained alcohol-related deaths, yet it remains underreported and poorly understood. This case report adds to the literature by presenting a middle-aged woman with a complex alcohol-related history who died within hours of hospital presentation, with autopsy findings consistent with SUDAM.

## Case presentation

Materials and methods

This is a retrospective case report based on clinical records and the post-mortem examination of a deceased female. Information was obtained from the electronic medical records, toxicology reports, and full autopsy findings.

Results

A 49-year-old woman presented to the emergency department via ambulance after her son reported a two day history of diarrhoea and vomiting resulting in slurred speech and confusion. Her past medical history included gallstones gastritis hypomagnesaemia, benign paroxysmal positional vertigo, oral thrush, anxiety, alcohol related chronic liver disease, multiple episodes of acute pancreatitis, chronic pancreatitis, gastro-oesophageal reflux disease, tinea corporis, migraines, alcoholism, menorrhagia, and irritable bowel disease. Prior to admission, the patient lived alone with support from social services. She mobilised using a wheelchair. She consumed approximately two bottles of wine per day for many years and smoked 30 cigarettes daily. She denied recent illicit drug use. On arrival, she was hypotensive and hypoglycaemic. She was managed for presumed gastroenteritis with intravenous fluids. Concerns regarding dark stools prompted consideration of endoscopy. Her hypoglycaemia was resistant to multiple treatments, including oral, intramuscular, and intravenous therapies. Fluid resuscitation produced only a mild improvement in hypotension. Blood gas analysis revealed metabolic acidosis with raised lactate and low bicarbonate. She became anuric with signs of fluid overload, suggesting renal failure. Due to instability, endoscopy and cross-sectional imaging were not performed. She continued to deteriorate and died later that night. Key laboratory values obtained prior to death are summarised in Table 1.

**Table 1:** Key laboratory values obtained prior to death ALT: alanine aminotransferase; AST: aspartate aminotransferase

Parameter	Result	Reference range
Blood glucose	1.8 mmol/L low	3.9 to 6.1 mmol/L
Sodium	129 mmol/L low	135 to 145 mmol/L
Potassium	3.2 mmol/L low	3.5 to 5.0 mmol/L
Magnesium	0.55 mmol/L low	0.7 to 1.0 mmol/L
Lactate	6.8 mmol/L high	< 2.0 mmol/L
Bicarbonate	15 mmol/L low	22 to 29 mmol/L
Creatinine	189 µmol/L high	45 to 90 µmol/L
Urea	15.2 mmol/L high	2.5 to 7.8 mmol/L
ALT	85 U/L high	< 40 U/L
AST	112 U/L high	< 40 U/L
Bilirubin	45 µmol/L high	< 21 µmol/L
Albumin	30 g/L	30 to 50 g/L

A post-mortem examination performed 19 days after death revealed no external evidence of jaundice trauma or significant disease. Internal examination found no significant cardiovascular or neurological pathology. Findings included cirrhosis consistent with alcoholic liver disease, sclerosed pancreas, pulmonary oedema, and bilateral pleural effusions. Toxicological analysis of femoral blood using tandem mass spectrometry showed a paracetamol concentration of 54.9 mg/L, which is above the therapeutic range but below toxic levels. Amitriptyline and nortriptyline were within therapeutic ranges. The presence of morphine, midazolam, loperamide, and ondansetron was consistent with hospital management. Ethanol was not detected in blood, and only trace amounts were found in urine, likely representing a post-mortem artefact. Probable causes of death included hypovolaemic shock secondary to fluid loss compounded by hepatic and renal dysfunction. Although drug levels were within therapeutic range, impaired hepatic metabolism may have potentiated their effects. No sepsis bleeding source or myocardial pathology was identified. The post-mortem certified cause of death as (a) multiorgan failure, (b) SUDAM, and (c) liver cirrhosis.

## Discussion

SUDAM refers to cases where an individual with a history of chronic or excessive alcohol consumption dies unexpectedly, and no clear anatomical or toxicological cause of death is identified at autopsy [2]. It is a recognised but poorly understood phenomenon that may represent a significant but under-appreciated contributor to alcohol-related mortality.

Epidemiological studies suggest SUDAM most often affects middle-aged adults (35-55 years) with long-standing heavy alcohol use [3]. Deaths are frequently unwitnessed and occur in domestic settings. Histopathological findings often demonstrate hepatic steatosis, gastritis, mild chronic pancreatitis, myocardial fibrosis, or renal tubular injury; however, none of these fully account for the fatal outcome [4].

Proposed mechanisms include cardiac arrhythmias, which may arise from electrolyte imbalances such as hypomagnesaemia and hypokalaemia, as well as from alcohol-induced myocardial changes. Autonomic dysfunction and QT prolongation further increase the risk of sudden cardiac death. In addition, metabolic disturbances, including severe hypoglycaemia and lactic acidosis, can contribute to physiological instability. Nutritional deficiencies, particularly thiamine deficiency, may also exacerbate both neurological and cardiac dysfunction [5].

**Figure 1:**
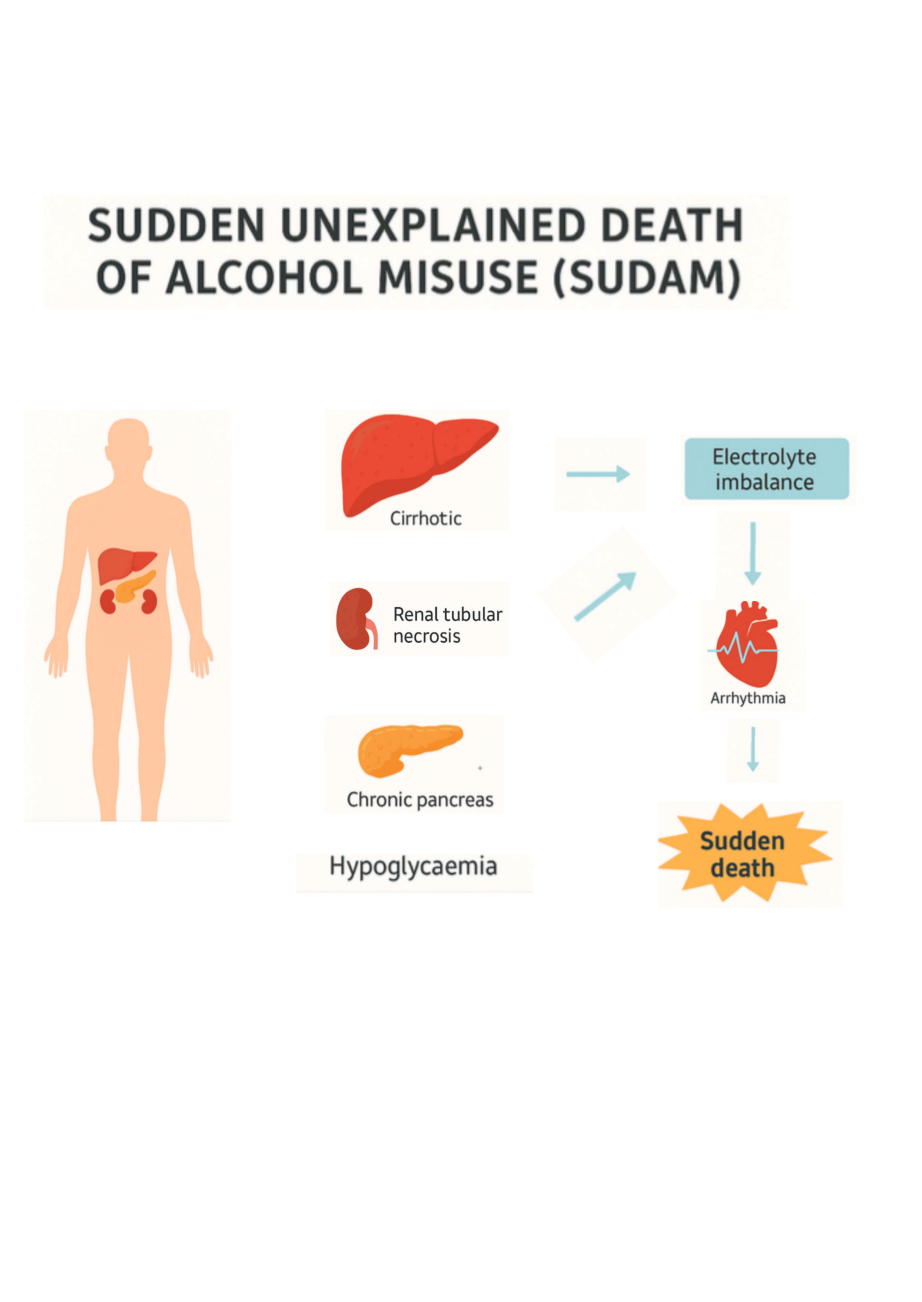
Proposed mechanisms leading to SUDAM SUDAM: sudden unexplained death of alcohol misuse This image has been created by the author

This patient demonstrated multiple risk factors for SUDAM, including chronic alcohol misuse, malnutrition, recurrent pancreatitis, and significant electrolyte and metabolic disturbances. Despite medical management, the rapid deterioration underscores the challenge of preventing fatal outcomes in this patient population. The schematic presented in Figure 1 provides a visual summary of the organ pathology and proposed mechanisms underlying this case.

Differential diagnoses include alcohol-related sudden cardiac death [2], seizure-related death (particularly during withdrawal) [2], Wernicke’s encephalopathy, alcoholic ketoacidosis, acute pancreatitis, and toxic ingestion (e.g., methanol or ethylene glycol), which was excluded by toxicology. SUDAM remains a diagnosis of exclusion, requiring comprehensive toxicological and histopathological investigation [2].

## Conclusions

This case illustrates the diagnostic challenges posed by sudden unexpected death in individuals with chronic alcohol misuse, particularly in the absence of a definitive anatomical cause. It underscores the importance of considering SUDAM as a potential diagnosis. A comprehensive post-mortem investigation, including histological and toxicological analysis, is essential to exclude alternative causes and support this exclusionary diagnosis. Greater awareness and recognition of SUDAM may improve the classification of alcohol-related mortality and inform preventive public health strategies targeting high-risk individuals.

## References

[1] (2018). Global status report on alcohol and health 2018. https://www.who.int/publications/i/item/9789241565639.

[2] Templeton AH, Carter KL, Sheron N, Gallagher PJ, Verrill C (2009). Sudden unexpected death in alcohol misuse-an unrecognized public health issue?. Int J Environ Res Public Health.

[3] Jones AW, Holmgren A (2003). Comparison of blood-ethanol concentration in deaths attributed to acute alcohol poisoning and chronic alcoholism. J Forensic Sci.

[4] Verrill C, Sheron N (2005). Alcohol-related harm-a growing crisis: time for action. Clin Med (Lond).

[5] Sorkin T, Sheppard MN (2017). Sudden unexplained death in alcohol misuse (SUDAM) patients have different characteristics to those who died from sudden arrhythmic death syndrome (SADS). Forensic Sci Med Pathol.

